# Overview of molecular mechanisms of plant leaf development: a systematic review

**DOI:** 10.3389/fpls.2023.1293424

**Published:** 2023-12-07

**Authors:** Zhuo Lv, Wanqi Zhao, Shuxin Kong, Long Li, Shuyan Lin

**Affiliations:** ^1^ Co-Innovation Center for Sustainable Forestry in Southern China, Nanjing Forestry University, Nanjing, China; ^2^ Bamboo Research Institute, Nanjing Forestry University, Nanjing, China; ^3^ College of Life Science, Nanjing Forestry University, Nanjing, China

**Keywords:** leaf primordium, leaf polarity, leaf size, leaf shape, leaf senescence

## Abstract

Leaf growth initiates in the peripheral region of the meristem at the apex of the stem, eventually forming flat structures. Leaves are pivotal organs in plants, serving as the primary sites for photosynthesis, respiration, and transpiration. Their development is intricately governed by complex regulatory networks. Leaf development encompasses five processes: the leaf primordium initiation, the leaf polarity establishment, leaf size expansion, shaping of leaf, and leaf senescence. The leaf primordia starts from the side of the growth cone at the apex of the stem. Under the precise regulation of a series of genes, the leaf primordia establishes adaxial-abaxial axes, proximal-distal axes and medio-lateral axes polarity, guides the primordia cells to divide and differentiate in a specific direction, and finally develops into leaves of a certain shape and size. Leaf senescence is a kind of programmed cell death that occurs in plants, and as it is the last stage of leaf development. Each of these processes is meticulously coordinated through the intricate interplay among transcriptional regulatory factors, microRNAs, and plant hormones. This review is dedicated to examining the regulatory influences of major regulatory factors and plant hormones on these five developmental aspects of leaves.

## 1 Introduction

Leaves serve as the primary photosynthetic organs in plants and are vital for capturing light energy and facilitating gas exchange. There are two types of simple and compound leaves, a leaf with only one leaflet on the petiole is referred to as a simple leaf, and its shape is determined by factors such as the tip, base, and margins of the leaf. Conversely, multiple single leaves (such as apical leaves, lateral leaves, and stipules) are collectively termed compound leaves. A typical leaf comprises three main parts: the blade, petiole, and stipules. Leaves are flat lateral structures that originate from the shoot apical meristem (SAM) (Zhang and Liu, 2022) and exhibit a high degree of morphological diversity (Tsukaya, 2014).

Leaf development is a complex, dynamic process that can be summarized into five processes: the first step is the leaf primordium initiation, the primordia begins in the peripheral zone of the apical meristem; the second step is the establishment of polarity, the initial leaf primordium develops in the direction of adaxial-abaxial growth axes, proximal-distal growth axes, medio-lateral growth axes; in the third step, the blade size is controlled, and the basic leaf shape is formed by the extension of the blade from the edge region of the adaxial plane and the abaxial plane; the fourth step is the regulation of leaf shape and leaf expansion to form the final shape (Yan et al., 2008; Xiong and Jiao, 2019; Wang et al., 2021a); the fifth step is leaf senescence which accompanied by programmed cell death (PCD) that occurs in plants (Tian et al., 2020)

Recently, the development of plant leaves has been explored from many angles such as anatomy, cytology and molecular biology, which makes the research more perfect. This review provides a review of the fundamental structure and developmental processes of leaves, along with an exploration of the molecular mechanisms involved in morphogenesis, leaf initiation, determination of leaf polarity, and the processes of leaf growth and senescence during development.

## 2 Initiation of leaf primordium

**Figure 1:**
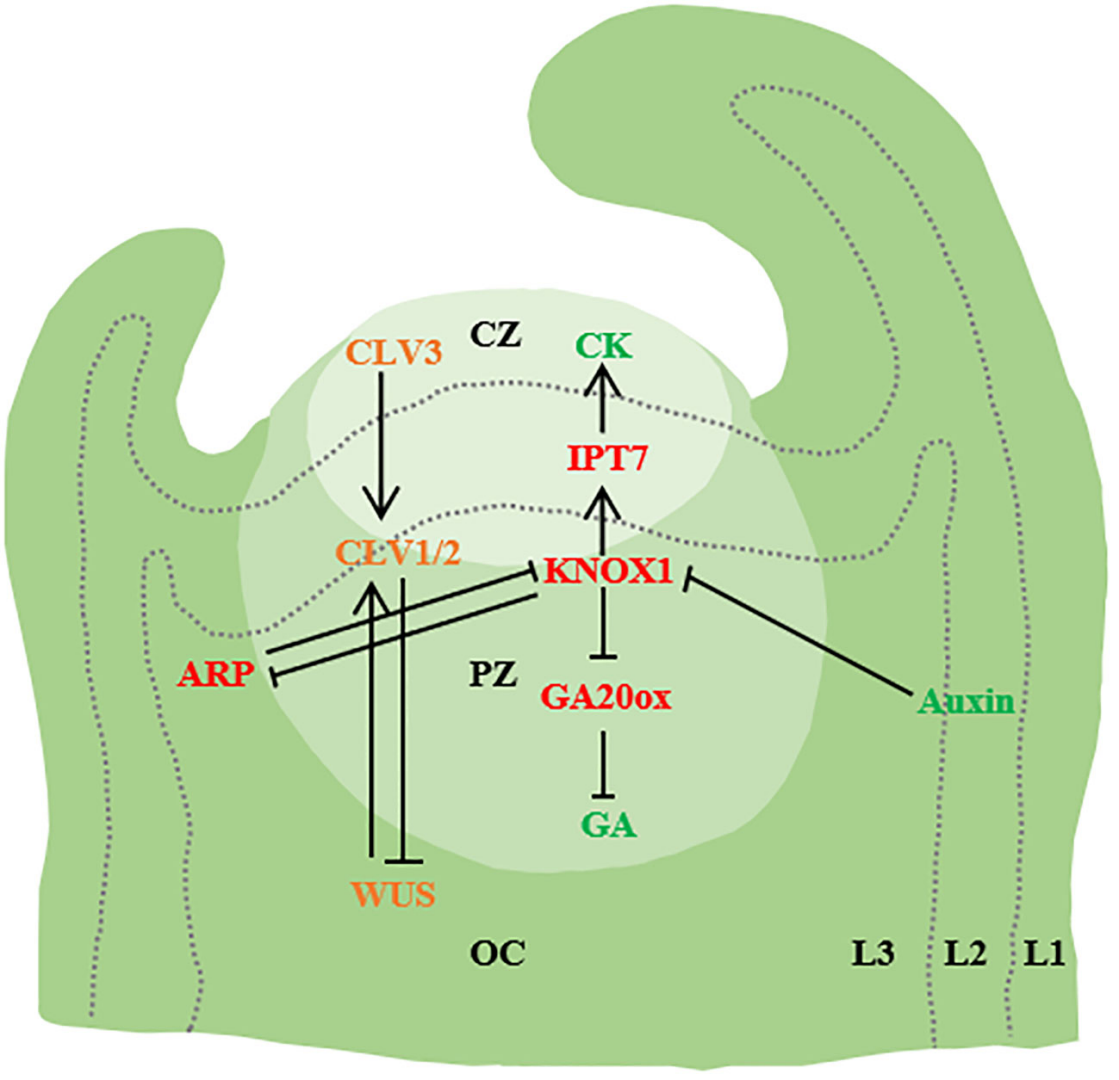
Initiation of leaf primordium. SAM is divided into three functional regions [central region (CZ), peripheral region (PZ) and costal region (OC)] and Layer1 (L1), Layer2 (L2) and Layer3(L3). WUS activates CLV3, and CLV3 further binds to CLV1/2, thereby inhibiting WUS expression. Auxin accumulation in the flanks of SAM through PIN1/AUX1 mediated polar transport triggers primordium development. In addition, KNOX1 maintains the role of stem cells, positively regulates CK, negatively regulates GA signaling through IPT7 and GA20ox, and ARF regulates the emergence of young primordiae (→ represents positive regulation, and T-shaped arrows represent negative regulation. The same below.) (Barkoulas et al., 2007; Bar and Ori, 2014; Kalve et al., 2014; Wang et al., 2021a).

The leaf primordia of plants start from SAM, which is an embryonal cell population with a “tunica-corpus” structure. The structure can be divided into three layers. The tunica cells divide into L1 and L2 cambium peripherally, and the corpus cells divide into L3 cambium peripherally or vertically. The L1 layer is the promeristem layer, which develops into the epidermis. L1 layer cells control the division pattern of their inner layer cells. The L2 layer is divided into three parts: the promeristem (part) develops into the lower epidermis, the protocambium develops into a vascular bundle, and the basic meristem layer (part) develops into cortical and medullary rays. Layer 3 is the basic meristem layer (part), which develops into a pith (**Figure 1**).

The shoot apical meristem (SAM) comprised by the central zone (CZ), the peripheral zone (PZ), and the organizing center (OC). At the apex of the SAM, there is a cluster of slowly dividing cells, constituting the central region of SAM. These cells are larger, possess stem cell functions, and play a pivotal role in maintaining the meristem’s integrity. The rate of cell proliferation and growth in this central region often differs significantly from that at the periphery. In the periphery of the central SAM region, cell division rates are notably accelerated. These rapidly dividing cells form the peripheral region of SAM, which serves as the origin for organ primordia such as leaf primordia. Below SAM lies the organizing center, also known as the Rib Meristem (RM), (Huang, 2003; Barton, 2010; Cao and Jiao, 2018; Xiong and Jiao, 2019). Within the SAM, the homeodomain transcription factor *WUSCHEL* (*WUS*) is expressed in the organizing center (OC) to uphold stem cells in the central zone (CZ). The migration of *WUS* to the central zone activates the accumulation level of the *CLAVATA3* (*CLV3*). CLV3 acts as a negative regulator by encoding a secreted peptide. This peptide triggers the transmembrane receptor kinase *CLV1* in the organizing center, resulting in the inhibition of WUS expression (Sassi and Vernoux, 2013).

During the formation of plant leaf primordium, the plant hormone auxin is the growth regulator of organ initiation. The highest local auxin concentration observed in the L1 cambium of the SAM. This localized increase in auxin concentration is facilitated by polarly localized PIN-FORMED1 (PIN1) efflux transporters. The rise in auxin levels coincides with the onset of leaf primordium formation, and the cellular response to auxin is mediated by AUXIN RESPONSE TRANSCRIPTION FACTORs (ARFs) (Ben‐Gera et al., 2012; Pinon et al., 2013; Bar and Ori, 2014; Dong and Huang, 2018; Wang et al., 2021). Auxin level has a negative effect on SAM size. Auxin transport of SAM in lateral organs can be inhibited by auxin transport switch, thereby maintaining SAM homeostasis and SAM size (Shi et al., 2018). AUX/PIN1 is involved in the regulation of simple leaf morphogenesis and compound leaf lobular initiation. Genes encoding AUXIN1 (AUX1) and LIKE-AUX1-2 (LAX2) are direct targets of auxin response transcription factor MONOPTEROS (MP) (Bhatia et al., 2016). Auxin also activates the expression of *PIN1* through MP, in addition, auxin activates the expression of cytokinin signaling inhibitor ARABIDOPSIS HISTIDINE PHOSPHOTRANSFER PROTEIN6 (AHP6) through MP, and AHP6 moves between cells to produce an inhibitory field that prevents premature growth of the primordium. AHP6 moves between cells to establish an inhibitory field that prevents premature primordium growth (Krogan et al., 2016; Du et al., 2018). In addition to auxin, cytokinin, another plant hormone, also plays a significant role in SAM maintenance (Werner et al., 2001; Kurakawa et al., 2007; Gordon et al., 2009; Shani et al., 2016).

Studies in *Arabidopsis thaliana*, *Zea mays* L., and *Antirrhinum majus* have unveiled two molecular mechanisms governing the initiation of leaf primordia (Barkoulas et al., 2007; Yan et al., 2008; Barton, 2010). The first mechanism involves the polar localization of the auxin transporter *PIN1*, ensuring the transport of auxin to the initial site of leaf primordium. Additionally, accumulated auxin inhibits the expression of the Class I *KNOTTED1*-like homeobox *(KNOX1)* gene (Reinhardt et al., 2003; Heisler et al., 2005; Jönsson et al., 2006; Wang et al., 2021a). The second mechanism is the mutual inhibition of the tip meristem maintenance gene KNOX1 and ARP[ASYMMETRIC LEAVES1(AS1)/ROUGH SHEATH2(RS2)/PHANTASTICA (ARP)] (**Figure 1**). In single-leaf species, the expression of the *KNOX1* in leaf primordium remains suppressed due to the inhibition of *ARP*, whereas in certain multiple-leaf species, reactivation of the *KNOX1* expression occurs and contributes to lobule formation (Byrne et al., 2000; Lodha et al., 2013; Wang et al., 2021). Apart from *ARP*, the *KNOX2* gene also promotes leaf development by counteracting *KNOX1* (Furumizu et al., 2015). Analogous to ARPs, heterotopic expression of *KNOX2* and its heterodimer partner BEL-LIKE HOMEODOMAIN (BELL) in the Arabidopsis relative *Cardamine hirsuta* inhibits SAM activity. During the development of compound leaves, *KNOX1* expression is reestablished within the leaf primordium, thereby initiating the formation of distinct lobules (Hay and Tsiantis, 2006; Leiboff et al., 2021).

## 3 Establishment of leaf polarity

**Figure 2:**
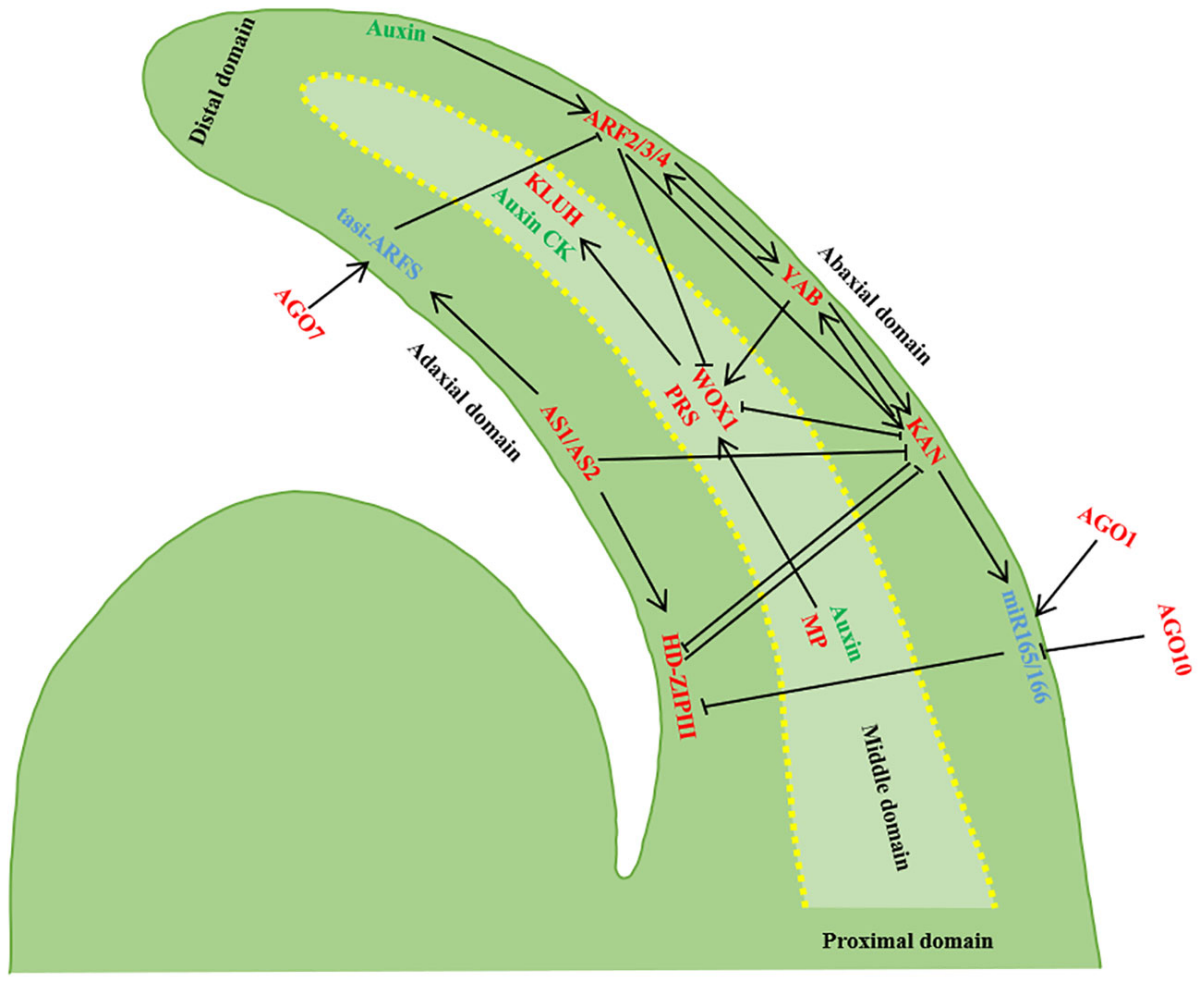
Establishment of leaf polarity. The developing leaf primordium has three domains, and the transcription factors in the three domains inhibit each other’s expression and control each other. Transcripts of AS1/AS2, HD-ZIPIII and tasi-ARF accumulate in the adaxial domain of the leaf primordium, transcripts of *ARF 3/ARF4*, *KAN* and miR165/166 accumulate in the abaxia domain, and *WOX1/PRS* are expressed in the intermediate domain of the leaf primordium. *AGO10* inhibits miR165/166, *AGO1* regulates miR165/166 and miR165/166 inhibits HD-ZIPIII. *AGO7* stabilizes ta-siR-ARF, and ta-siR-ARF degrades of ARF2/3/4. tasi-ARF and miR165/166 Rnas can move between cells and inhibit *ARF2/3/4* and HD-ZIPIII after transcription, and ARF2/3/4 is controlled by auxin. *KAN* and HD-ZIPIII antagonized each other, and *ARF2/3/4* and *KAN* were inhibited by *AS1/AS2*. *KAN* inhibited the expression of *WOX1* and *PRS*, while *WOX1* and *PRS* inhibited the expression of *KAN*. Adaxial expression of *MP* and off-axes enrichment of auxin together localized *WOX1* and *PRS* expression in the intermediate domain. In addition, *MP* may be the direct target of positively expressed HD-ZIPIII, and *YAB* promotes the expression of *WOX1/PRS* with *KAN* and *ARF2/3/4*, while *YAB* promotes the expression of *WOX1/PRS* (Barkoulas et al., 2007; Kalve et al., 2014; Du et al., 2018; Wang et al., 2021a).

During leaf development, polarity establishment is an important process affecting leaf morphology. When leaf primordium emerges from the edge of the meristem, the three axes that determine the polar growth of leaf morphogenesis, namely, adaxial-abaxial axes (front-back of the leaf), proximal-distal axes (base-tip of the leaf) and medio-lateral axes (main vein-edge of the leaf), have been determined (Conklin et al., 2019; Muszynski et al., 2020). The development and morphogenesis of leaves along these three axes are regulated by plant genetic mechanisms and various environmental factors (Wang et al., 2021). The regulatory mechanism is shown in **Figure 2**.

### 3.1 Adaxial-abaxial polarity

The near-distal axes, also referred to as the adaxial-abaxial axes, plays a pivotal role in establishing blade polarity and determining blade thickness. Palisade tissue develops on the adaxial plane of leaves, while spongy tissue forms on the abaxial plane. The maintenance of adaxial-abaxial polarity in the leaf primordium is determined by a complex gene regulatory network, while the characteristics of the adaxial plane and abaxial plane contribute to gene antagonism, thereby enhancing stability in maintaining two distinct cell fates. Adaxial-abaxial polarity is influenced by transcription factors, small RNAs, and auxin. The determination of adaxial-abaxial cell fate occurs before the basal rise of leaf primordium and is mediated by AS2 and KANADI1 (KAN1). The positional information established by the AS2-KAN1 prepattern transforms the non-polar distribution of auxin within the leaf primordium into a polar distribution, relying on ARF-dependent auxin signal transduction to establish adaxial-abaxial polarity (Burian et al., 2022). The adaxial-abaxial axes play a role in defining medio-lateral axes polarity by controlling the differential distribution of auxin and its downstream signaling molecules in the leaves, facilitating flat leaf growth (Qi et al., 2014). There is a partial overlap between auxin and the downstream response factor MP, resulting in a high auxin signal in the intermediate region between the adaxial-abaxial axes plane. Adaxial expression of MP directly activates the accumulation level of *WUSCHEL-LIKE HOMEOBOX1* (*WOX1*) and *PRESSED FLOWER* (*PRS*), which are key determinants in the formation of the intermediate zone necessary for lateral blade expansion (Barkoulas et al., 2007; Kalve et al., 2014; Wang et al., 2021). Downstream auxin pathway response factors *ARF2*, *3*, and *4* specifically expressed on the abaxial plane of leaves inhibit *WOX1* and *PRS* expression in that domain. Regulatory genes expressed in the abaxial domain of the leaf exert a suppressive effect on the genes expressed in the adaxial domain. The combined action of *MP* and *ARF2/3/4* enables specific *WOX1* and *PRS* expression in the middle leaf domain, facilitating leaf unfolding (Shani et al., 2006; Barkoulas et al., 2007; Kalve et al., 2014; Guan et al., 2017; Wang et al., 2021a).

Adaxial polarity genes encompass AS1, AS2, and the Class III Homeodomain-leucine Zipper (*HD-ZIPIII*) family genes. The AS2 genes encode plant-specific LATERAL ORGAN BOUNDARIES (LOB) domain proteins. Research in *Arabidopsis thaliana* has revealed that *HD-ZIP III* is a pivotal gene for the development of the leaf adaxial plane. Genes within the *HD-ZIPIII* family, including *PHABULOSA* (*PHB*), *PHAVOLUTA (PHV)*, and *REVOLUTA (REV)*, work in concert to regulate the expression and differentiation of SAM cell division toward the leaf’s adaxial plane (Zhong and Ye, 1999; McConnell et al., 2001; Reinhart et al., 2002; Emery et al., 2003; Juarez et al., 2004). Mutations in *PHB*, *PHV*, and *REV* can lead to leaf abnormalities. The AS1-AS2 complex positively regulates *HD-ZIPIII*, inhibits the abundance of *KNOXI* and *YABBY*, and facilitates the establishment of adaxial plane polarity in leaves (Jönsson et al., 2006). The fate of leaf abaxial plane cells is governed by the *KAN* and *YAB* gene families, various ARFs, and small RNA molecules such as microRNAs and trans-acting short-interfering RNAs (ta-siRNA). The *KANs* encode proteins harbored with GARP domain, expressed in the distal domain, and they mutually inhibit the *HD-ZIPIII* expression, influencing the properties of the adaxial plane while regulating *YAB* gene expression (Kerstetter et al., 2001). The *YABs* playing a vital role in polarity maintenance and leaf development. The ARF family genes encode transcription factors responsive to plant auxin. All *PHB*, *PHV*, and *REV* genes have complementary sequences with miR165 and miR166 (Rhoades et al., 2002; Tang et al., 2003). Ectopic expression of microRNAs (miR165/166) inhibits the adaxial plane gene *HD-ZIP III*, resulting in leaf is apaxial (Otsuga et al., 2001; Emery et al., 2003; Juarez et al., 2004).

### 3.2 Proximal-distal polarity

The length of the leaves is determined by the proximal-distal axes, and *BLADE-ON-PETIOLE1* (*BOP1*), *BOP2*, *ROTUNDIFOLIA3* (*ROT3*), *ROT4*, *LONGIFOLIA1* (*LNG1*), and *LNG2* participate in proximal-distal axes formation (Kerstetter et al., 2001; Ha et al., 2003; Ha et al., 2004; Hepworth et al., 2005). The single mutant *bop1* and double mutant *bop1* and *bop2* both exhibited leaf growth on the petiole with a reduced petiole region (Ha et al., 2003; Hepworth et al., 2005). The expression of *BOP1/2s* is localized to the basal and adaxial regions of the leaf primordium, exerting influence on leaf cell fate by inducing *AS2* expression and inhibiting *KNOXI* (Ha et al., 2003; Jun et al., 2010). Moreover, the polarity of the proximal-distal axes is also under the regulation of auxin. Investigations conducted on *Arabidopsis* leaves have unveiled the roles of ARF6 and ARF8, two pivotal transcription factors within the auxin signal transduction pathway, in promoting proximal-distal growth of leaf reproductive organs, such as stem leaves and sepals. ARF6 and ARF8 instigate the synthesis and signal transduction of BRs by activating the expression of *DWARF4 (DWF4)*, a pivotal enzyme gene in BR synthesis. BRs, in turn, facilitate the demethylation of cell wall pectin, resulting in isotropic in-plane cell wall loosening. By modulating BR biosynthesis, auxin influences cell wall mechanics and guides cell-oriented growth, ultimately giving rise to leaves with diverse shapes and overseeing the proximal-distal growth of leaf reproductive organs (Xiong et al., 2021).

### 3.3 Medio-lateral polarity

The medio-lateral axes determine the width of the leaf, and the WUSCHEL‐LIKE HOMEOBOX(WOX) family is involved in the establishment of the medio-lateral axes of the leaf, which promotes the growth of the medio-lateral axes while inhibiting the growth of the adaxial-abaxial axes. The WOX family can be divided into three branches: ancient branch, intermediate branch, and modern branch. WOX transcription factors belong to the homeodomain superfamily and have typical DNA-binding domains (Bürglin and Affolter, 2016). The conserved function of *WOX1* is regulating the development of medio-lateral axes of leaves. Phylogenetic analysis of the entire WOX family in *Solanum lycopersicum* found that knocking out the *SlLAM1*, a modern branch of the WOX family, through CRISPR/Cas9-mediated genome editing resulted in a narrowing of the leaves and a reduction in the number of lobules (Wang et al., 2021c). Two different WOX transcription factors STF and WOX9 in *Medicago truncatula* and *Nicotiana tabacum* jointly regulate the expression of cytokinin oxidase gene CKX3 in plants through antagonistic effects. This regulation influences leaf development by affecting cytokinin content and cell division. (Wang et al., 2022a). *M. truncatula STENOFOLIA(STF*) and *N. tabacum LAMINA1*(*LAM1)* are homologous genes of *AtWOX1*, and *stf/lam1* mutants show leaf narrowing phenotype. In addition, overexpression of *STF* in rice (*Oryza sativa)*, *Brachypodium distachyon*, and the energy grass *Panicum virgatum*, respectively, showed the characteristics of wider leaves and thicker stems (Tadege et al., 2011; Wang et al., 2017). The leaf narrowing phenotype of *stf/lam1* mutants can be restored to varying degrees by modern branch members of the WOX family (*WUS*, *WOX1-WOX6*), but the intermediate branch member *WOX9* aggravates the leaf phenotype of *stf/lam1* mutants, and studies have found that *STF* directly inhibits the expression of *WOX9* (Lin et al., 2013; Wolabu et al., 2021). The recessive wide leaf mutant *wl1*(*wide leaf 1*), a novel *DROUGHT AND SALT TOLERANCE*(*DST*) allele, has also been found in rice. *WIDE LEAF1(WL1)* interacts with Tillering and Dwarf1(TAD1), a coactivator of anaphase-promoting complex/cyclosome (APC/C) multi-subunit E3 ligase and is degraded by the APC/C^TAD1^ complex *via* the ubiquitin/26S proteasome degradation pathway. In addition, *WL1* further recruits Histone Deacetylase HDAC to inhibit the expression of narrow leaf gene *NARROW LEAF1*(*NAL1)* by binding to rice TPR-like transcriptional corepressors, thereby regulating leaf width (You et al., 2022).

In addition, leaf polarity establishment is under the regulation of ARGONAUTE (AGO) proteins and long non-coding RNAs (lncRNAs). AGO proteins belong to the RNA-binding protein class and play a pivotal role in small RNA-mediated gene silencing (Baumberger and Baulcombe, 2005). In Arabidopsis thaliana, *AGO1* and *AGO10 (PINHEAD/ZWILLE)* are part of the same clade. *AGO1* primarily governs the miRNA pathway and contributes to the post-transcriptional gene silencing of transgenes (Baumberger and Baulcombe, 2005), *AGO10* maintains undifferentiated stem cells in stem meristem tissue (Liu et al., 2009b). *AGO1*, in conjunction with miR165/166 and tasiR-ARF, facilitates target cleavage (Mi et al., 2008), and the mutation of *ago1* resulted in the lateral organs with adaxial and abaxial defects (Lynn et al., 1999). AGO10 inhibits miR165/166 expression in SAM and modulates the establishment of adaxial-abaxial polarity in leaves. The mutation of *ago10/pnh/zll* exhibit abnormal increases accumulation level of *miR165/166* in leaves and SAM, leading to a repression of *HD-ZIP III* transcripts (Liu et al., 2009; Zhu et al., 2011). lncRNAs also play a significant role in leaf development. Studies in *Liriodendron chinense* have identified various lncRNA-transcription factor (TF) regulatory modules, including *lch-lnc6026-BLH2*, *lch-lnc0809-ATHB4*, *lch-lnc4261/5500-GRF1*, l*ch-lnc5465-bHLH30*, and *lch-lnc2601/3202/6972-TCPS*, *lch-lnc1857/4867/6438-AUX/IAAs*. A variety of lncRNA-TF regulatory modules are involved in the establishment of leaf polarity and the regulation of leaf morphology (Tu et al., 2022). In rice, endogenous lncRNA TWISTED LEAF (TL) is transcribed by another strand of the R2R3-MYB coding locus *OsMYB60*. Down-regulating *TL* through RNA interference (RNAi) or overexpressing *OsMYB60* can lead to leaf distortion in transgenic rice, underscoring the crucial role of lncRNAs in maintaining the flatness of rice leaves (Liu et al., 2018).

## 4 Regulation of leaf size

**Figure 3:**
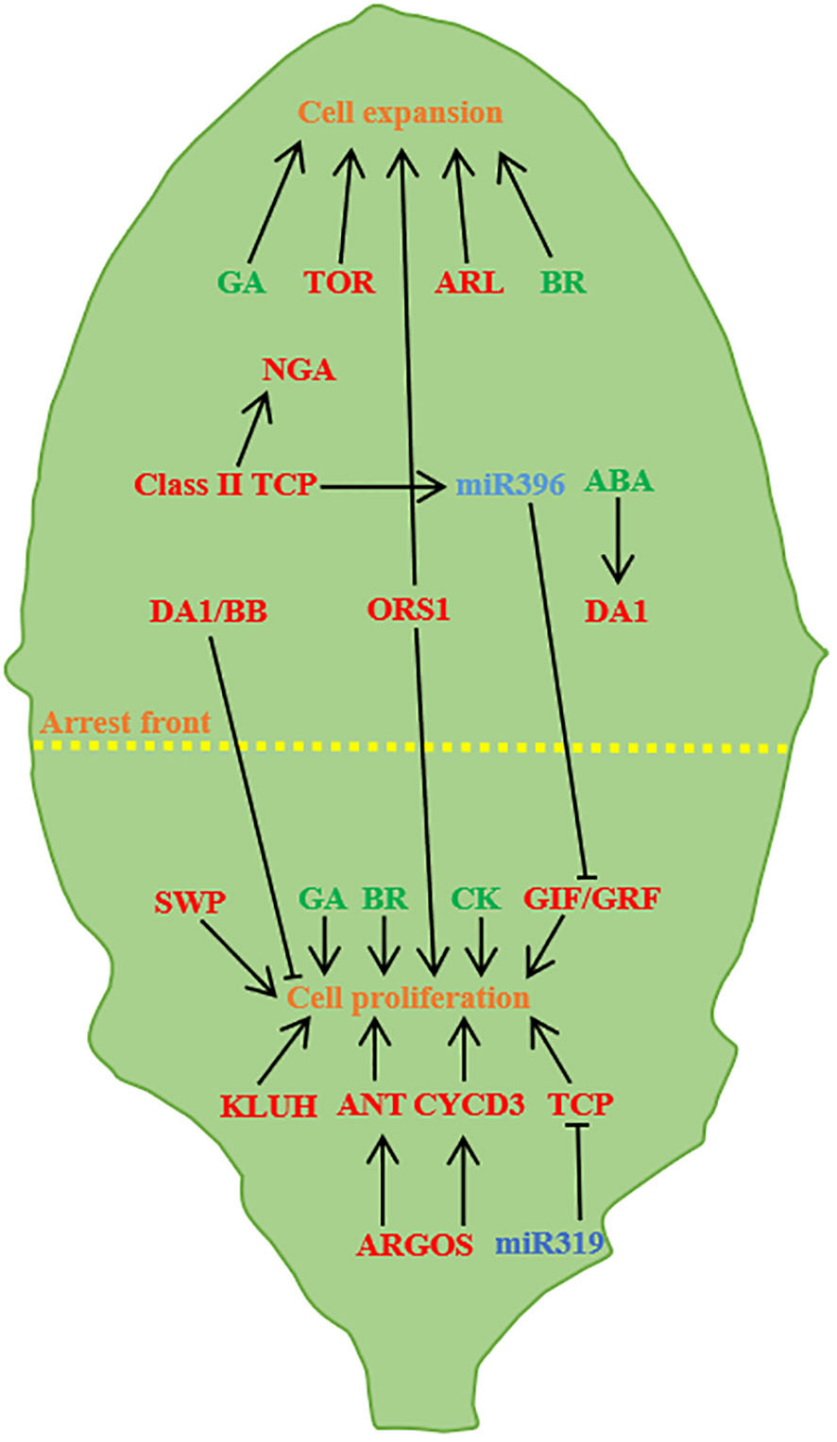
Regulation of leaf size (A case study of *Arabidopsis* leaves). Leaf size is controlled by cell proliferation and cell expansion. ARGOS promotes cell proliferation through ANT and CYCD3, and miR319 negatively regulates TCP and GRF/GIF transcription factors to promote proliferation. TOR and ARL promote cell expansion, TCP activates the *NGA* to promote cell expansion, BB and DA1 control the timing of proliferation, and abscisic acid partially facilitates the transition by regulating DA1. KLUH, SWP and CK promote cell proliferation, ORS1, GA and BR have positive effects on cell proliferation and cell expansion (Barkoulas et al., 2007; Kalve et al., 2014; Du et al., 2018; Wang et al., 2021a).

As the leaf primordium starts and its polarity is established, the leaf primordium begins to expand, forming its final size, under the regulation of plant hormones (auxin, gibberellin, cytokinin and brassinolide), microRNA [miR319-TEOSINTE BRANCHED, CYCLOIDEA and PCF1/2(TCP) and miR396-GROWTH‐REGULATING FACTOR(GRF) regulatory units] and *APETALA2/ETHYLENE RESPONSIVE FACTOR*(*AP2/ERF*) family gene. The regulatory pattern is shown in **Figure 3**.

### 4.1 Regulation of leaf size by plant hormones

Auxin promotes cell proliferation and expansion (Leyser et al., 1996) and acts as a signal during cell proliferation to determine the shape and size of the final organ (Lincoln et al., 1990). The reverse regulatory balance of the two auxin-regulated genes *AUXIN* -*GENE INVOLVED IN ORGANAIZE* (*ARGOS*) and *ARF2* is necessary for auxin to function properly in cell division, cell expansion and differentiation. Auxin can induce the expression of *ARGOS* genes, causing plants to exhibit larger leaves than normal *via* regulating or maintaining the transcription of *AINTEGUMENTA*(*ANT*) (Mizukami and Fischer, 2000; Hu et al., 2003; Spartz et al., 2012). *ARF2* inhibits cell proliferation through the ANT pathway, and loss of *ARF2* function promotes cell proliferation, resulted in leaf size increasing. Cytokinin (CK), gibberellin (GA) and brassinolide (BR) can also regulate leaf size. Both GA and BR promote leaf growth by promoting cell proliferation and expansion. Lack of GA and BR or insensitive mutants will lead to smaller leaves, and overexpression of GA and BR will make leaves larger (Li et al., 2001; Richards et al., 2001; Mitchum et al., 2006; Cheon et al., 2010; Oh et al., 2011; Zhiponova et al., 2013). CK manipulating the expression of *IPT* or *HvCKX2* genes, thereby regulating cell proliferation mode and leaf size (Černý et al., 2013; Skalák et al., 2019). Studies on rice have found that CK is involved in the formation of rice plant type, CK accumulation mediated by CK *OXIDASE/DEHYDROGENASE3* (*OsCKX3*) in rice controls the development of leaf pillow and negatively regulates the angle of leaves, and the loss of *OsCKX3* can induce the asymmetric growth and development of leaf pillow (Huang et al., 2023).

### 4.2 Effects of genes on leaf size during leaf development

During leaf growth and development, TCPs play critical roles. Based on sequence differences in conserved domains, it can be divided into two subgroup including classI (promoting cell proliferation and plant growth) and class II (inhibiting cell proliferation) (Liu and Gao, 2016; Yang et al., 2019). Specifically, miR396 targets *TCP* genes, including *TCP2, TCP3, TCP4, TCP10*, and *TCP24*, which regulate cell proliferation (Palatnik et al., 2003). The *tcp* mutants increase leaf size and curvature by up-regulating cyclin-encoding genes (Schommer et al., 2008; Bresso et al., 2018). miR319 primarily regulates TCP transcriptional regulators and high miR319 expression causes severe leaf development defects, resulting in large and wrinkled leaves (Palatnik et al., 2003). Moreover, ERF4 interacts with TCP15, a key activator of the mitotic cell cycle, to promote intracellular replication by inhibiting *CYCA2;3* expression. This interaction positively regulates cell enlargement and leaf expansion. The high abundance of *TCP15* in young leaves promotes cell mitosis and proliferation, while *ERF4* was highly expressed in mature leaves, and it essential for endokaryotic replication and cell expansion (Ding et al., 2022).

TCP family *DE1CINCINNATA-LIKE TEOSINTE BRANCHED1/CYCLOIDEA/PROLIFERATING CELL FACTORS* (*CIN-TCP*) redundancy of transcription factors inhibits growth, CIN-TCP is co-regulated by miR319 after transcription, and mutations in CIN-TCP or ectopic expression of miR319 result in larger leaves (Palatnik et al., 2003). *TCP4* is the target of miR319 (Palatnik et al., 2003), in *A. thaliana*, point mutations in the miR319 target sites of *TCP4* reduce interactions with miR319, resulting in higher levels of miR396, lower accumulation level of *GRFs*, and smaller leaf formation (Rodriguez et al., 2010). Overexpression of *miR319* in *Arabidopsis jaw-D* mutants leads to decreased expression of *TCP2*, *3*, *4*, *10*, and *TCP24* and the formation of larger leaves (Palatnik et al., 2003).

*GRF* family regulates cell proliferation in a redundant manner, promotes leaf growth and development, and controls leaf size (Wang et al., 2020; Wu et al., 2021). Plants carrying GRF-mutated genes have small and narrow leaves, and *GRF* overexpressors tend to form excessively large leaves (Kim et al., 2003; Horiguchi et al., 2005; Kim and Lee, 2006). *Arabidopsis GRF* produces different leaf phenotypes, and overexpression of *AtGRF1*, *AtGRF2*, *AtGRF3*, or *AtGRF5* results in larger leaves than normal leaves (Kim et al., 2003; Horiguchi et al., 2005; Debernardi et al., 2014). Mutants *atgrf 1/2/3*, *atgrf 3*, *atgrf 4*, or *atgrf 5* lead to smaller and narrower leaves than normal leaves (Kim et al., 2003; Horiguchi et al., 2005; Kim and Lee, 2006; Lee et al., 2009; Debernardi et al., 2014). *GRF9* restricts cell proliferation during leaf growth by controlling *ORG3* expression (Omidbakhshfard et al., 2018). GRF transcription factors and GRF-INTERACTING FACTOR (GRF-GIF) transcriptional coactivators worked together to positively regulate leaf development. *Arabidopsis* GIF family consists of *GIF1* (*ANGUSTIFOLIA3*, *AN3*), *GIF2* and *GIF3*, which are positive regulators of cell proliferation. GIF family genes form functional complexes with GRF transcription factors and participate in cell proliferation activities during leaf development (Kim and Kende., 2004; Lee et al., 2009; Debernardi et al., 2014; Lee and Kim., 2014). The expression levels of *CsGRF* and *CSGIF1* were high in young leaves of tea (*Camellia sinensis*), and the expression levels gradually decreasing with the increase of leaf permanent maturity, indicating that *CsGRF* and *CSGIF1* genes may essential for early leaf tissue formation (Wu et al., 2017). In maize (*Zea mays* L.), *ZmGRF10* regulates leaf size by restricting cell proliferation, and overexpression of *ZmGRF10* can lead to impaired cell proliferation and reduced leaf length (Wu et al., 2014). In addition to the collaboration between GRFs and GIFs, the *GRF* is regulated by miR396 in the leaf development regulatory network (Debernardi et al., 2014). *TCP* can regulate miR396, while miR396 targets *GRF* (Huang et al., 2019), and overexpression of miR396 inhibits *GRF*, resulting in reduced cell number, smaller leaves, repression of miR396 or overexpression of *GRF* induces plants to grow larger leaves (Liu et al., 2009a; Rodriguez et al., 2010; Wang et al., 2011). GRFs are the upstream repressor of *KNOX*, and *Hordeum vulgare* BGRF1 can be used as the suppressor of the intron sequence of *Hooded/Barley Knotted3* (*Bkn3*) of *KNOX* (Kuijt et al., 2014). However, *AtGRF4*, *5*, and *6* can inhibit *KNOTTED-LIKE FROM ARABIDOPSIS THALIANA2* (*KNAT2*) promoter activity (Kuijt et al., 2014).

Transcription levels of GRFs are affected by gibberellinic acid (GAs), GA3 in rice increased and decreased the expression levels of six (*OsGRF1*, *OsGRF2*, *OsGRF3*, *OsGRF7*, *OsGRF8*, *OsGRF10*, and *OsGRF12*) and one (*OsGRF9*) *OsGRF* gene, respectively (Choi et al., 2004). (Choi et al., 2004). In Chinese cabbage (*Brassica rapa* ssp. *pekinensis*), the transcript levels of most *BrGRF* are induced by exogenous GA3 treatment (Wang et al., 2014). In oilseed rape (*Brassica napus*), the expression level of *BnGRFs* under GA treatment may be negatively regulated (Ma et al., 2017). In peach (*Prunus persica*), the expression of six *PpGRF* genes (*PpGRF1*, *PpGRF4*, *PpGRF5*, *PpGRF6*, *PpGRF7*, and *PpGRF*10) was up-regulated and three *PpGRF* genes (*PpGRF*2, *PpGRF6*, and *PpGRF7*) were down-regulated after GA3 treatment (Liu et al., 2022). All CsGRF genes of sweet orange (*Citrus sinensis*) are involved in leaf development. After treatment with GA3, the transcription levels of *CsGRF5* and *CsGRF6* did not change, while the transcription levels of the remaining seven CsGRF genes showed significant improvement (Liu et al., 2016).

AP2/ERF family genes are unique transcription factors found in plants, playing pivotal roles in processes such as plant growth, hormone-induced development, ethylene response, and stress response. In *L. chinense*, three AP2 genes - *LcERF94, LcERF96*, and *LcERF98* - are predominantly associated with early leaf development and morphogenesis, exhibiting high expression levels in both SAM and leaf primordia (Zong et al., 2021). Notably, overexpression of the AP2/ERF transcription factor *BOLITA (BOL)* in *Arabidopsis* and *Tobacco* results in reductions in both cell size and number, consequently yielding smaller leaves (Marsch-Martinez et al., 2006). Additionally, *SsAP2/ERFs*, exhibits widespread expression in mature sugarcane leaves, indicating its significant role in the growth and development of sugarcane (Li et al., 2020). Leaf size is intricately regulated by a multitude of genes, with microRNAs binding to AGO proteins to guide mRNA cleavage or suppress the translation of complementary RNAs. This targeted regulation of non-protein-coding transcripts by miRNAs can stimulate the generation of tasiRNA populations (Mallory et al., 2008). *ARF3/ETTIN (ETT)* and *ARF4* are among the targets of *TAS3* ta-siRNA, governing normal leaf development through the action of AGO7/ZIPPY, and, reciprocally, *TAS3* ta-siRNA inhibits ARF3/4 expression (Adenot et al., 2006). *ANT*, a regulator in the AP2 family, emerges as a pivotal regulator of final leaf size. Overexpression of *ANT* leads to enlarged leaves, whereas *ANT* mutants yield smaller leaves (Mizukami and Fischer, 2000). In A. thaliana, the regulation of *ANT* on organ size is modulated by *ARGOS*; the loss of *ARGOS* function leads to smaller leaves. In plants overexpressing *ARGOS*, the loss of *ANT* function inhibits the macro-leaf phenotype (Hu et al., 2003). The DA1 regulatory factor encodes ubiquitin receptors that determine the organ size by limiting the cycle of cell proliferation. Point mutations in ubiquitin receptors result in the formation of larger leaves. Mutations in the phenotypic enhancer of *da1-1 (EOD1)/BIG BROTHER (BB)* in *da1* mutants also caused in larger leaves (Li et al., 2008). Cytochrome *P450 KLUH (KLU)/CYP78A5* acts as a stimulator of plant organ growth. Loss function of *KLU* lead to smaller organs due to premature cessation of cell proliferation. Conversely, *KLU* overexpression results in larger organs with an increased number of cells (Anastasiou et al., 2007).

In *Arabidopsis*, the growth is positively correlated with the expression level of TARGET OF RAPA-MYCIN (TOR) kinase. Decreased or increased gene expression in TOR leads to decreased or increased organ and cell size, respectively (Deprost et al., 2007). Overexpression of *ARGOS-LIKE (ARL)* leads to the enlargement of cotyledons, leaves, and other lateral organs in Arabidopsis (Hu et al., 2006).

## 5 Regulation of leaf shape

**Figure 4:**
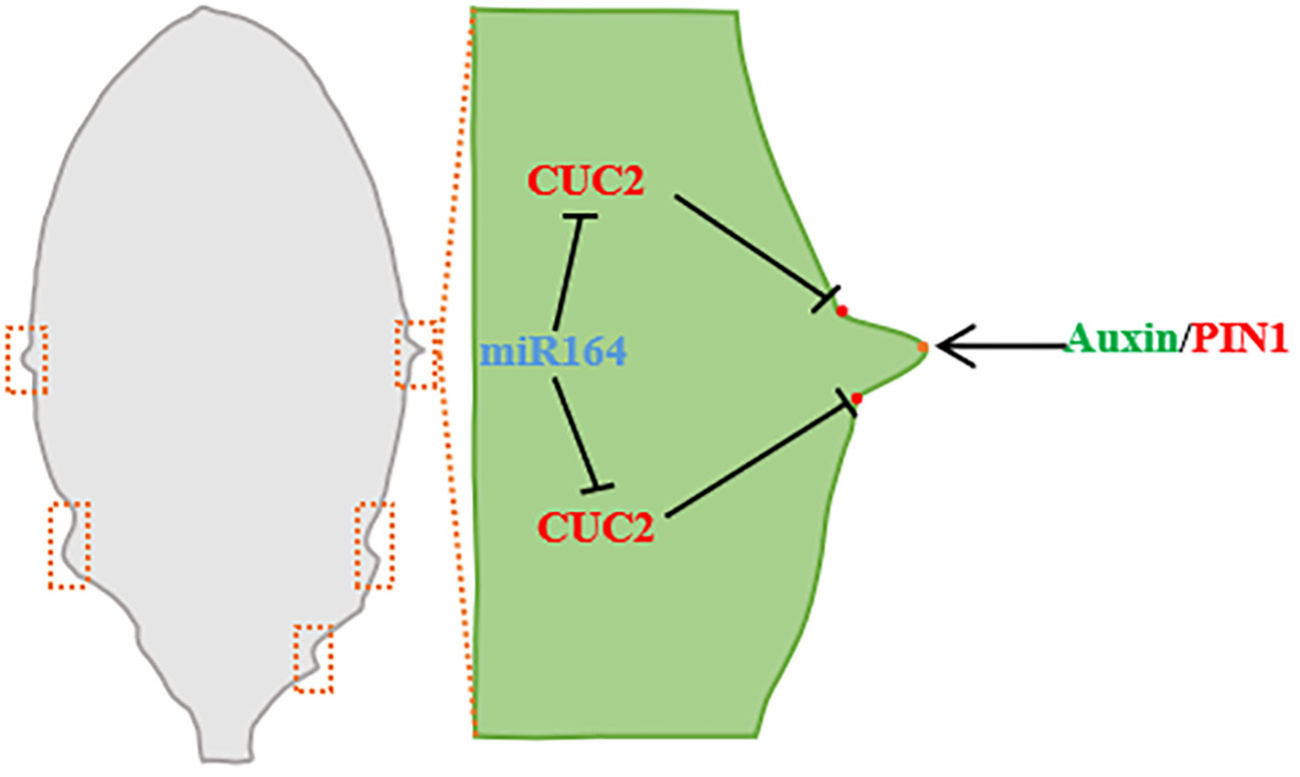
Leaf edge development (taking *Arabidopsis* leaf as an example). During leaf margin development, miRNA164 and CUC2 are expressed in the overlapping regions of serrated sagging, CUC2 promotes the establishment of PIN1 convergence points, and PIN1 convergence points produce the maximum value of auxin along the serrated tip of leaf margin. Auxin maximously inhibits CUC2 at the tip of the tooth and promotes the growth of the tooth (Barkoulas et al., 2007; Wang et al., 2021a).

The shape of leaves varies significantly both within and between plant species, ranging from slender to oval. Leaf shape diversity is primarily driven by variations along the basic-apical axes, with further adjustments leading to a rich spectrum of leaf forms, which may include serrations, notches, and lobes predominantly situated along the leaf margins. Leaf shape is predominantly governed by the growth behaviour of the marginal blastozone, which possesses meristematic capabilities (**Figure 4**).

### 5.1 Regulation of plant hormones on leaf shape during leaf development

Many components of the auxin signaling pathway influence the formation of a compound leaf or leaf margin serration. Auxin regulates cell division by inducing the expression of *AS1* and *AS2*, thereby regulating leaf shape, and mutants of *as1* and *as2* genes exhibit a distinct bilateral asymmetrical growth pattern, resulting in the formation of lobes and lobular structures from the petioles (Semiarti et al., 2001). As a member of the Aux/IAA family, the ENTIRE expression in the intermediate domain between the *S. lycopersicum* lobules inhibited the auxin response to maintain the leafless character on the axes. The ENTIRE interaction with ARF activators SIARF19A, SIARF19B, and SIMP, and ARF activation dosed to promote lobular growth to varying degrees (Xiong and Jiao, 2019). The NAC gene *GOBLET*(*GOB*) can interact with auxin, and its activity alters auxin signal distribution and, together with the ENTIRE auxin response, promotes compound leaf formation (Xiong and Jiao, 2019). The effect of GA on plant leaf shape development varies according to different species. Increasing the content of GA in single-leaf species can form leaf shapes with smooth leaf margins and no engraving, and GA can also promote the simplification of compound leaves (Achard et al., 2009; Bar and Ori, 2014). In leaf development, GA and CK play an antagonistic role. The activity of GA is related to KNOX1 and TCP. TCP can increase the GA level, while KNOX1 protein can reduce the GA level and increase the CK level. This interplay between the two hormones helps regulate the balance between them, ultimately controlling the development of leaf margin morphology. (Li et al., 2007).

### 5.2 Effects of genes on leaf shape during leaf development

Leaf shape traits are the outcome of complex gene networks, with key regulators like *KNOX* and *REDUCED COMPLEXITY* (*RCO*) homeobox genes playing pivotal roles in shaping leaf complexity and contributing to the evolutionary diversification of leaf morphology (Hay and Tsiantis, 2010; Vlad et al., 2014). The diversity in leaf geometry arises from two distinct processes. Firstly, the *SHOOTMERISTEMLESS* (*STM*) of the *KNOX* family enhances overall organ growth relative to leaf margin patterning, resulting in the formation of lobes. Secondly, *RCO* suppresses local growth in the vicinity of developing lobes, accentuating the differences in leaf shape stemming from the establishment of marginal patterns. Both processes are significant in *Cardamine occulta* but not in *Arabidopsis* leaves (Kierzkowski et al., 2019). KNOX1 are pivotal in maintaining undifferentiated cell fates and contributing to the development of complex leaf primordia in the shoot apical meristem (SAM). Their activity is suppressed by AS1-AS2 complexes, which are encoded by transcription factors that inhibit the expression of the *KNOX1* in *Arabidopsis* leaves. Elevated levels of *KNOX1* and *RCO* in *as1* and *as2* mutants collaboratively determine the formation of compound leaves. *KNOX1* is expressed at the leaf midvein base, while *RCO* is symmetrically expressed at the base of lobular primordia. Both *KNOX1* and *RCO* curtail local cell growth by extending the growth potential of leaf primordium cells, promoting anisotropic cell expansion, and giving rise to compound leaves (Wang et al., 2022c). *KNOX1* expression is indispensable for the development of lobules in plants with compound leaves. The coordination between *LATE MERISTEM IDENTITY1 (LMI1)* and *KNOX1* governs leaf development, resulting in a diverse range of leaf shapes, such as broad, shallow, and compound leaves, depending on the combinations of these genes (Chang et al., 2019). Leaf margin notches in plants are closely associated with *KNOX1* expression, observed only in species featuring leaf margin notches (Rast-Somssich et al., 2015). Within the *KNOX1* family, *STM*, *BREVIPEDICELLUS (BP)*, and *KNOTTED-1 (KN1)* exert significant influence on the development of leaf margin shapes. Additionally, ARP genes and miRNAs are involved in regulating leaf shape development, with *ARP* down-regulation altering the number and shape of lobular lobes, while miR396 overexpression modulates leaf shape development by targeting the *GRF* genes (Jia et al., 2015).

The MYB gene family regulates leaf shape, and the MYB transcription factor has a highly conserved MYBDNA-BD (MYB domain). In plants, MYB proteins have multiple subfamilies, such as MYB1R, R2R3-typeMYB, MYB3R, and 4R-MYB factors, which are key factors in the regulatory network controlling growth, development, metabolism, and stress response (Huang et al., 2013). *Nicotiana benthamiana PHANTASTICA* (*NbPHAN*) is a novel R2R3-type MYB gene in *Tobacco*, *NbPHAN* silencing inhibits the expression of *NTH20* gene, The leaves showed severe downward curling and abnormal leaf growth along the main vein (Huang et al., 2013). MYB transcription factor *CLAUSA*(*CLAU*) in *S. lycopersicum* promotes leaf morphogenesis by reducing cytokinin signal, CLAU facilitates differentiation by suppressing cytokinin (CK) signaling, while CK promotes morphogenesis by inhibiting CLAU expression and suppressing morphogenetic potential to regulate leaf development. Partly by weakening CK signaling (Bar et al., 2016). *Trifoliate* (*Tf*) gene in *S. lycopersicum* regulates leaf morphology, and *Tf* encodes the transcription factor R2R3MYB and the transcription factor LATERAL ORGAN FUSION1(LOF1) and LOF2 associated with *Arabidopsis*, *Tf* is expressed in leaf margins and leaf axils, *Tf* maintains leaf morphogenesis by inhibiting cell differentiation, *Tf* mutation leads to a single narrow leaf during the early stage of leaf development in tomato, and a pair of lateral lobules in the terminal and long petioles in the later stage (Naz et al., 2013). The *OsMYB103L* in rice encodes the R2R3-MYB transcription factor, and overexpression of *OsMYB103L* leads to leaf curl (Yang et al., 2014).

The interaction of auxin and NO APICAL MERISTEM/CUP-SHAPED COTYLEDON (NAM/CUC) transcription factors is involved in the regulation of leaf margin model (Žádníková and Simon, 2014). Reduced function of NAM/CUC inhibits the growth of leaf margins and leads to lobular reduction and fusion (Blein et al., 2008). NAM/CUC transcription factors promote the formation of leaf margin nicks in single leaves and regulate lobular differentiation and separation in compound leaves, and the silencing of NAM/CUC transcription factors leads to simplification of leaf shape (Bar and Ori, 2014). *CUC* inhibits growth between leaf notch and lobule (Hasson et al., 2011), and promotes the growth leaf teeth (Kawamura et al., 2010). The transcriptional levels of many leaves’ development regulatory genes, including CUC family genes, are regulated by NGALs, and the regulatory effects of NGALs on gene transcription are primarily negative and dependent on CUC2 (Shao et al., 2020). *Arabidopsis* NGAL1 can directly bind to the promoter of CUC2 and suppress the expression of CUC2 (Shao et al., 2020). In *Arabidopsis*, ectopic expression of CUC1 triggers lobular formation, CUC2 acts on the downstream of NGALs to regulate the lobed/serrated leaves, and the inactivation of CUC3 inhibits the serrated part (Kawamura et al., 2010; Hasson et al., 2011). In the B3 family NGATHA-LIKE (NGAL) subfamily of *Arabidopsis*, overexpression of the three transcription factors NGAL1-3, respectively, can lead to the formation of cup-shaped cotyledon and smoot-edged true leaves. *ngaltri*, a trimutant with NGAL1-3 function loss, showed an enhanced dentate leaf margin phenotype, indicating that NGALs were involved in leaf margin development (Shao et al., 2020). In addition, Zeng et al. (2022) studies on *Citrus reticulata* Blanco leaves also found that CiKN1 and CiKN6 have a significant impact on citrus leaf morphological development, CiKN1 and CiKN6 regulate the molecular mechanism of citrus leaf development, and they can combine with each other to form complexes. By binding to the miR164a promoter, it inhibits the expression of CimiR164a, thereby playing a role in the regulation of leaf development through the miR164a-CUC2 pathway (Zeng et al., 2022).

DNA methylation mediated by DNA methyltransferases (DMT) is an important epigenetic modification widely present in plant genomes, which regulates the shape of leaves during the development of leaves, and also controls cell division and expansion. In *Populus simonii*, the expression of DMF144 and DMF143 in the genomic regions of *PtHT1* and *PtHT2* was found to be higher in young leaves compared to mature leaves. This suggests that their expression may be suppressed by DNA methylation after cell division and expansion are completed, resulting in the differentiation of leaf shape in natural populations (Ci et al., 2016). DMF40, a methylation marker associated with leaf circumference in *P. simonii*, is located in the promoter region of *PtPIN1*, is situated within the promoter region of *PtPIN1*. It exerts a pronounced inhibitory effect on *PtPIN1* and likely plays a crucial role in leaf development within natural populations (Ci et al., 2016). The transcriptional activity of plants is related to DNA methylation on the genome. There is a correlation between genomic methylation and histone H3.3 (H3 variant) enrichment in *A.thaliana*, knockdown of H3.3 will lead to various phenotypic defects and dysregulation of response genes. H3.3 knockdown results in serrated leaf margins (Wollmann et al., 2017).

In their recent study on rice, Zhou et al. (2023) discovered that OsSNF7.2, a subunit of the ESCRT-III complex in rice, interacts with the auxin biosynthesizer OsYUC8. This interaction plays a crucial role in regulating rice leaf curl by affecting the transport and endosomal degradation of OsYUC8. SERRATE (SE) serves as a core protein in both miRNA biogenesis and mRNA selective splicing. Another intriguing finding comes from Chen et al. (2023), who identified SEAIRa as an antisense intragenic lncRNA transcribed from the 3′end of *SE*. SEAIRa functions by inhibiting *SE* expression, resulting in serrated leaf margins. Research on maize leaves, as reported by Satterlee et al. (2023), revealed the role of the rim domain in leaf primordium edge plane growth. Genetically redundant WOX3 transcription factors regulate this rim domain, and high-order mutations of *Wox3* genes in maize result in a significant reduction in leaf width and disruption of leaf growth and patterning. The gene expression signature linked to proximal-distal polarity in the initial lingual supports the concept that both lobes and lingua exhibit dorsoventral asymmetry and grow functionally through the juxtaposition of paraxial and distal domains. In Moso bamboo, the *PheLBD29* (*lateral organ boundaries domain 29*) was highly expressed in leaves. Overexpression of *PheLBD29* in *Arabidopsis* resulted in small and backward-curved leaves, with 35S:PheLBD29 *Arabidopsis* leaves exhibiting a transformation of cells from the positive side to the backside (Wu et al., 2023). The modification of N6-methyladenosine (m6A) in mRNA is a critical regulator of gene expression and plant growth and development. Huang et al. emphasized the significance of Vir-like m6A methyltransferase-associated (VIRMA) as a scaffold that connects the catalytic core components of the m6A methyltransferase complex. In their investigation of upland cotton (*Gossypium hirsutum*), they observed widespread expression of the genes *GHVIR-A* and *GHVIR-D* of VIRMA in various tissues. Disruption of the expression of *GhVIR* genes had a notable influence on the size, shape, and total cell number of leaf cells, thereby influencing the morphogenesis of cotton leaves (Huang et al., 2022).

## 6 Leaf senescence

**Figure 5:**
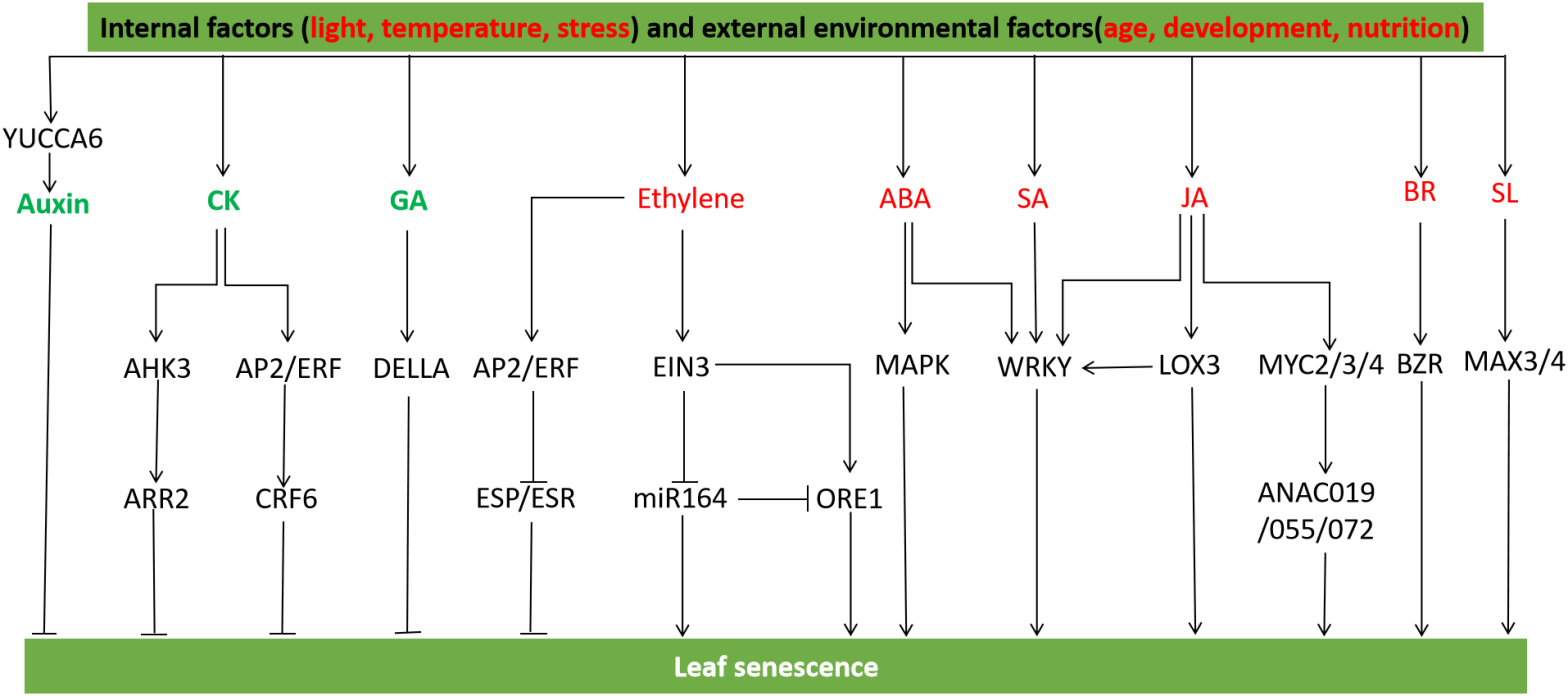
Hormonal and gene regulation of leaf senescence. YCCA6 regulates auxin biosynthesis and inhibits leaf senescence. The AP2/ERF transcription factor CRF6 mediated by cytokinin inhibits senescence, and the cytokinin receptor AHK3 regulates leaf senescence through the regulatory factor ARR2. The abnormal accumulation of DELLA protein delayed leaf senescence by blocking GA biosynthesis. Ethylene activated AP2/ERF gene to regulate leaf senescence, ERF transcription factor inhibited the expression of ESP/ESR, a negative regulator of leaf senescence, *ESR* knockout promoted leaf senescence, while *ESR* overexpression did the opposite. EIN3 activates ORE1 and NAP to positively regulate leaf senescence. EIN3 inhibits miR164 transcription and up-regulates the transcription level of *ORE1/NAC2*, the target gene of miR164. ABA induces WRKY transcription factor and promotes early senescence of leaves under dark treatment. SA treatment induces SAGs expression, and WRKY influences plant aging and defense signaling pathways in SA-mediated signaling cascades. WRKY interacts with JA biosynthesis gene *LOX3* to promote leaf senescence, MYC2/3/4 protein can activate JA-induced chlorophyll degradation, and the signaling pathway of MYC2/3/4 and NAC protein ANAC019/055/072 induces leaf senescence. BRs activates BZR family transcription factors to promote leaf senescence. SL genes *MAX3* and *MAX4* accelerate leaf senescence (Mayta et al., 2019; Guo et al., 2021a).

Leaf senescence is a degenerative process that occurs during the final stage of leaf development, and it is regulated by a combination of internal factors (such as age, development, and nutrition) and external environmental factors (including light, temperature, and stress) (Tian et al., 2020). During leaf senescence, the expression profile of a large number of genes is changed, which forms a complex genetic regulatory network with other signal regulatory pathways in transcriptional regulation (Lim et al., 2007). Leaf senescence includes several processes such as chlorophyll decomposition and macromolecular degradation, at the appropriate age, plant leaves begin to senescence and decompose lipids, proteins, nucleic acids, and carbohydrates, thereby retransporting nutrients in the senescent leaves to seeds, storage organs or other growing tissues (Lim et al., 2007; Yu et al., 2021). The functional and regulatory interaction networks of many molecular components during leaf senescence vary with the degree of senescence (Lim et al., 2007) (**Figure 5**).

### 6.1 Regulation of plant hormones on leaf senescence

Plant hormones play a crucial role in the regulation of senescence. Among them, ethylene, brassinosteroids (BRs), jasmonic acid (JA), salicylic acid (SA), abscisic acid (ABA), and strigolactone (SLs) promote senescence, while auxin, gibberellins (GAs), and cytokinins delay senescence (Breeze et al., 2011; Guo and Gan, 2012; Jibran et al., 2013).

In the ethylene signaling pathway, key factors such as EIN3, miR164, and NAC (NAM, ATAF, and CUC) transcription factors like ORE1/NAC2, form regulatory networks that mediate leaf senescence (Qiu et al., 2015). *EIN3*, *ORE1*, and *CCGs* participate in ethylene-mediated chlorophyll degradation during leaf senescence in *Arabidopsis*. Specifically, *EIN3* acts as a positive regulator of *CCG* expression in this process (Kim et al., 2009; Qiu et al., 2015). Ethylene can activate *AP2/ERF* and regulate the senescence of plant leaves, ERFs *AtERF4* and *AtERF8* which belongs to Class II increase with plant senescence, and the enhanced expression of *NtERF3*, *AtERF4*, or *AtERF8* can lead to premature senescence of transgenic *Arabidopsis* leaves. *terf4-terf 8* double mutants delay leaf senescence (Koyama et al., 2013). ERF transcription factors inhibiting leaf senescence occurs negative regulatory factors EPITHIOSPECIFIER PROTEIN/EPITHIOSPECIFYING SENESCENCE REGULATOR (ESP/ESR) gene expression (Miao and Zentgraf, 2007). *ESR* knockout can accelerate the senescence rate of leaves, and *ESR* overexpression leaves can slow down the senescence rate (Miao and Zentgraf, 2007). The mitogen-activated protein kinase (MAPK) cascade is a crucial signal transduction pathway in eukaryotic cells, consisting of three components: MAPK, MAPK kinase (MAPKK), and MAPKK kinase (MAPKKK). In ABA signaling, MAPKKK18 and MAPKKK are involved, with MAPKKK18 regulating plant senescence through ABA-dependent protein kinase activity, thereby controlling plant growth and the timing of senescence (Matsuoka et al., 2015). *TaWRKY7* is induced by ABA, and *TaWRKY7* overexpression can prevent water loss in leaves and enhance tolerance to drought, while *TaWRKY7* expression is up-regulated during the senescence process of *Arabidopsis* leaves, promoting the early senescence of leaves under dark treatment, overexpression of *TaWRKY7* leads to premature senescence of transgenic *Arabidopsis* leaves (Zhang et al., 2016b). The additional application of salicylic acid (SA) induces the expression of senescence-associated genes (SAGs), while the WRKY transcription factor *AtWRKY70* plays a role in influencing plant senescence and defense signaling pathways within salicylic acid-mediated signaling cascades. The *AtWRKY70* mutation results in the upregulation of developmental senescence-associated genes (SAGs) and defense genes in the salicylic acid (SA) (*PR1* and *PR2*) and jasmonic acid/ethylene (JA/ethylene) (*COR1* and *PDF1.2*) signaling pathways. This implies a connection between plant defense mechanisms and the developmental programs associated with leaf senescence (Ulker et al., 2007). *WRKY53* serves as a positive regulator of senescence, while *WRKY70* acts as a negative regulator of senescence. The interaction among *WRKY53*, *WRKY54*, *WRKY70*, and *WRKY30* plays a role in regulating leaf senescence, facilitating the integration of the regulatory network involving internal and environmental signals during this process (Besseau et al., 2012). BRs activate BZs transcription through BRI1 receptor-like kinases and their well-defined signal transduction pathways, and *WRKY6* in the WRKY family inhibits its own promoter activity as well as that of closely related WRKY family members, *WRKY6* can regulate the expression of senescence-related genes in the senescence process (Robatzek and Somssich, 2002). WRKY transcription factor TaWRKY40-D is the promoter of leaf aging in transgenic *Arabidopsis*, and *Arabidopsis* plants overexpressed with *TaWRKY40-D* lead to premature leaf senescence after JA and ABA treatment (Zhao et al., 2020b). Senescence-related genes of *Arabidopsis* are up-regulated in *wrky57* mutants, and auxin can antagonize the JA-induced leaf senescence process through WRKY57, in addition, JA down-regulates WRKY57 protein levels, while auxin up-regulates WRKY57 protein levels (Jiang et al., 2014). The WRKY transcription factor aWRKY42-B, a member of the WRKY family, actively participates in both developmental and dark-induced leaf senescence. TaWRKY42-B facilitates leaf senescence by interacting with the JA biosynthetic gene AtLOX3 and its homolog TaLOX3, thus promoting the accumulation of JA content (Zhao et al., 2020a). During dark treatment, the plant hormone ethylene significantly induced strigolactone biosynthesis genes *MORE AXIALLY GROWTH3*(*MAX3*) and *MAX4*, it showed that strigolactone was synthesized in leaves during senescence (Ueda and Kusaba, 2015).

*YUCCA* encodes flavin-containing monooxygenases (FMO), facilitating the hydroxylation of the amino group of tryptamines (Zhao et al., 2001). With the increase of free IAA concentration, *YUCCA6*-activated mutants *yuc6-1D* and *35S:YUC6 Arabidopsis* plants exhibit delayed senescence and reduced expression of senescence-associated genes (SAGs) in leaves (Kim et al., 2011). In the process of leaf senescence, the removal of DELLA protein inhibition results in premature leaf senescence, while the enhancement of DELLA protein delays leaf senescence. The removal or enhancement of DELLA protein leads to the upregulation or downregulation of *SAG12* and *SAG29*. After DELLA inhibition was removed, the mutant *ga1-3 gai-t6 rga-t2 rgl1-1 rgl2-1* (abbreviated as Q-DELLA/ga1-3) exhibited premature leaf senescence, while the mutant ga1-3, which blocks GA biosynthesis and accumulates abnormal DELLA protein, delayed leaf senescence (Chen et al., 2014). The cytokinin-mediated AP2/ERF transcription factor Cytokinin response factor6 (CRF6) has a negative regulatory effect on leaf developmental senescence (Zwack et al., 2013). There are three cytokinin receptors in *Arabidopsis*, namely AHK2, AHK3 and AHK4/CRE1WOL. AHK3 plays a significant role in regulating leaf lifespan through the regulatory factor ARR2. Missense mutations occurring in the extracellular domain of AHK3 have been observed to delay leaf senescence (Kim et al., 2006).

### 6.2 Regulation of genes during leaf senescence

Leaf senescence is regulated by a variety of genes, and age-dependent leaf senescence is an important research focus, mainly focusing on annual and perennial plant leaf senescence. Studies on age-dependent leaf senescence in annual plant *Arabidopsis* found that AtWDS1 (encoding WD repeat protein), as a novel REDOX homeostasis regulator, negatively regulates age-dependent and dark-induced leaf aging (Fu et al., 2019). The *AtFer1* ferritin isoform is functionally implicated in processes leading to age-dependent senescence in *Arabidopsis* (Murgia et al., 2007). The ACBP3 (acyl-CoA binding protein) transgenic line exhibited age-dependent leaf senescence (Lee et al., 2011). The *ACBP3* (acyl-CoA binding protein) transgentic line showed age-dependent leaf senescence (Xiao et al., 2010). The *oresara9* (*ore9*) mutant leaves of *Arabidopsis* ORE9 prolong life during the age-dependent natural aging process by delaying the onset of various aging symptoms(Woo et al., 2001). The genome-wide H3K9 acetylation level of rice flag leaves increased with age-dependent aging, and the density and width of acetylated lysine residue 9 of histone H3 (H3K9ac) were positively correlated with gene expression and transcription elongation (Zhang et al., 2022b). Senescence-associated NAC (Sen-NAC) regulates the aging of autumn leaves of perennial poplar (*Populus tomentosa*). Age-dependent increases in intron retention (IR) splicing variants from Sen-NAC can fine-tune the molecular mechanisms of *Populus* leaf senescence (Wang et al., 2021b).

In addition, leaf senescence is also regulated by various transcription factors such as SQUAMOSA promoter binding protein (SBP), WRKY, C2H2, NAM/ATAF/CUC (NAC), bZIP, APETALA2 (AP2), MYB, etc. (Eulgem et al., 2000; Chen et al., 2002; Lin and Wu, 2004; Buchanan-Wollaston et al., 2005). NAC transcription factors (ANAC019, AtNAP, ANAC047, ANAC055, ORS1, and ORE1) are potential downstream elements of ETHYLENE INSENSITIVE2 (EIN2). EIN3, as a downstream signaling molecule of EIN2, binds to *ORE1* and *AtNAP* promoters, stimulating their transcription. Consequently, EIN3 positively regulates leaf senescence by activating *ORE1* and *AtNAP* (Li et al., 2013; Kim et al., 2014). EIN3 acted on ORESARA2 (ORE2)/ORE3/EIN2 downstream, inhibited *miR164* transcription and up-regulated *miR164* target gene *ORE1/NAC2* transcription, *miR164* overexpression or *ORE1/NAC2* knockdown inhibits EIN3-induced early-senescence phenotype (Li et al., 2013). The expression of miR164 is negatively regulated by EIN2 and gradually decreases with age, resulting in upregulated expression of ORE1 (Kim et al., 2009). SUPPRESSOR OF OVEREXPRESSION OF CO1 (SOC1) serves as a trans-regulator of Pheophytinase (PPH). SOC1 functions by inhibiting dark-induced leaf chlorophyll degradation and senescence, achieved through its negative regulation of Pheophytinase *PPH* expression in *Arabidopsis* (Chen et al., 2017). In the Basic helix-loop-helix (bHLH) subgroup IIIe, factors such as MYC2/3/4 proteins activate Chlorophyll catabolic genes (CCGs), facilitating JA-induced chlorophyll degradation. Furthermore, downstream of MYC2/3/4 protein, three NAC family proteins, ANAC019/055/072, directly promote the expression of *CCGs* (*NYE1/SGR1*, *NYE2/SGR2*, and *NYC1*) during chlorophyll degradation (Zhu et al., 2015). Overexpression of NAM transcription factor *BnaNAM* induces ROS production and leaf chlorosis in Rapeseed (*Brassica napus*) and positively regulates leaf senescence (Wang et al., 2022b). The WRKY transcription factor plays a crucial role in various aspects of plant physiology, including plant defense against pathogens, response to stress conditions, regulation of leaf senescence, and facilitation of plant growth and development. The overexpression of *GhWRKY17* in cotton in *Arabidopsis* upregulates senescence related genes *AtWRKY53*, *AtSAG12*, and *AtSAG13*, and accelerates senescence in *Arabidopsis* leaves (Gu et al., 2018). WRKY gene *CpWRKY71* in *Chimonanthus praecox* and *CpWRKY71* transgenic plants showed premature leaf senescence (Huang et al., 2019). *Leaf senescence1*(*LS1*) gene encoding C2H2-type zinc finger protein, *ls1* mutants will lead to premature senescence of leaves and decrease of chlorophyll content, and the expression of *LS1* in young leaves is lower than that in mature and senescent leaves, destruction of *LS1* function can promote ROS accumulation, accelerated leaf senescence, and cell death in rice (Zhang et al., 2022a). The C2H2-type zinc finger transcription factor MdZAT10 in Apple (*Malus domestica*) has been found to play a significant role in leaf senescence. It increases the expression of genes associated with senescence and promotes the acceleration of leaf aging. Additionally, MdZAT10 has the ability to enhance the transcriptional activity of MdABI5 on *MdNYC1* and *MdNYE1*, leading to an accelerated leaf senescence process. This highlights the important regulatory role of MdZAT10 in controlling and modulating leaf senescence in Apple (Yang et al., 2021).

MYB-type transcription factors play a critical role in regulating plant growth and development, as well as in response to various abiotic stresses. Specifically, the expression of MYBH, a homologous gene of the MYB transcription factor MYBS3 in *Arabidopsis*, has been found to be upregulated in both aged leaves and leaves subjected to darkness treatment. Overexpression of MYBH has been shown to result in premature leaf senescence, while the *MYBH* mutant *mybh-1* exhibited a delayed onset of plant senescence. Moreover, overexpression of *MYBH* has shown to enhance the expression of *SAUR36*, a key regulator of auxin that promotes leaf senescence. This phenomenon further accelerates leaf senescence induced by ABA and ethylene, highlighting the role of *MYBH* in mediating the senescence process (Huang et al., 2015). The findings have shed light on the intricate regulatory mechanisms by which MYB transcription factors, exemplified by MYBH, orchestrate plant responses to environmental cues and developmental processes, ultimately affecting leaf senescence (Zhang et al., 2011). The expression of the MYB-related transcription factor, *Oryza sativa* RADIALIS-LIKE3 (OsRL3), has been observed to be up-regulated in isolated leaves undergoing dark-induced senescence. This up-regulation of *OsRL3* contributes to the promotion of leaf senescence process (Park et al., 2018). Under both dark and ABA-induced leaf senescence conditions, the knockout mutant of *osmyb102* accelerated the senescence of rice, and overexpression of *OsMYB102* controls the expression of SAGs (Piao et al., 2019). When MYBR1 is overexpressed under the control of *OxMYBR1*, leaf senescence is delayed, and plants with *mybr1* gene function loss show faster chlorophyll loss and senescence (Jaradat et al., 2013).

## 7 Peroration

The regulatory network of leaf development has been gradually established and improved, and the development of leaves in more and more plants is continuously explored. Research on species is no longer limited to the single-leaf model plant *Arabidopsis*, and the leaf development process of compound-leaf model plants like tomatoes is also receiving increasing attention. The regulation of compound-leaf development is known to be incredibly intricate, with the involvement of various transcription factor families such as WOX, TCP, and MYB playing a pivotal role in the process in tomato plants. Leaf development itself is a complex and variable phenomenon, influenced by interactions between multiple transcription factors and hormones that ultimately govern leaf growth. Understanding these dynamics has been a central focus of research pertaining to leaf development models. One crucial aspect impacting leaf growth patterns is the establishment of an auxin concentration gradient facilitated by the auxin transport protein PIN1. Additionally, a class of KNOX genes acts as the primary genetic determinant in this process. Specifically, the KNOX1 protein regulates the balance between GA and CK during leaf development, and the inhibition of KNOX1 protein is necessary to drive forward the progression of leaf development.

In recent years, the single-cell transcriptome has allowed researchers to obtain a large amount of transcriptomic, epigenetic, and proteomic information from single cells, making spatial quantitative measurement of gene expression abundance possible. Spatial transcriptome can correlate the gene expression information of cells with their spatial location information. This makes it possible for us to study the developmental trajectories of various cells in the process of leaf development. The combination of single-cell transcriptome sequencing and spatial transcriptome sequencing is advantageous and complementary. It allows us to simultaneously obtain information on individual cell heterogeneity and the structural location of cells in tissue space. The combination of time and space multidimensional research techniques provides a method for in-depth understanding of the heterogeneity between cells in the process of leaf growth and development and for analyzing various growth phenomena during cell development.

## Data availability statement

The original contributions presented in the study are included in the article/supplementary material. Further inquiries can be directed to the corresponding authors.

## Author contributions

ZL: Investigation, Writing – original draft. WZ: Investigation, Writing – review & editing. SK: Investigation, Writing – review & editing. LL: Investigation, Writing – review & editing. SL: Resources, Supervision, Writing – review & editing.
